# Myxobacteria in high moor and fen: An astonishing diversity in a neglected extreme habitat

**DOI:** 10.1002/mbo3.464

**Published:** 2017-04-11

**Authors:** Kathrin I. Mohr, Tanja Zindler, Joachim Wink, Elke Wilharm, Marc Stadler

**Affiliations:** ^1^ Microbial Drugs Helmholtz Centre for Infection Research Braunschweig Germany; ^2^ Department of Supply Engineering Ostfalia Wolfenbüttel Germany; ^3^ Microbial Strain Collection Helmholtz Centre for Infection Research Braunschweig Germany

**Keywords:** biodiversity, bioprospecting, microbial ecology, *Myxococcales*, phylogeny

## Abstract

Increasing antibiotic resistances of numerous pathogens mean that myxobacteria, well known producers of new antibiotics, are becoming more and more interesting. More than 100 secondary metabolites, most of them with bioactivity, were described from the order *Myxococcales*. Especially new myxobacterial genera and species turned out to be reliable sources for novel antibiotics and can be isolated from uncommon neglected habitats like, for example, acidic soils. Almost nothing is known about the diversity of myxobacteria in moors, except some information from cultivation studies of the 1970s. Therefore, we evaluated the myxobacterial community composition of acidic high moor and fen both with cultivation‐independent 16S rRNA clone bank analysis and with cultivation. Phylogenetic analyses of clone sequences revealed a great potential of undescribed myxobacteria in high moor and fen, whereby all sequences represent unknown taxa and were detected exclusively by cultivation‐independent analyses. However, many clones were assigned to sequences from other cultivation‐independent studies of eubacterial diversity in acidic habitats. Cultivation revealed different strains exclusively from the genus *Corallococcus*. Our study shows that the neglected habitat moor is a promising source and of high interest with regard to the cultivation of prospective new bioactive secondary metabolite producing myxobacteria.

## 1 Introduction

Myxobacteria are soil inhabiting, Gram‐negative *Deltaproteobacteria* and are distributed all over the world (Dawid, 2000). Due to their nutritional requirements, they can be divided in predators and cellulose‐degraders. Beside their interesting life‐style expressed by the development of often colorful fruiting bodies and dry resistant myxospores (Reichenbach & Dworkin, 1992), myxobacteria are extremely interesting with regard to their great potential to produce a high diversity of bioactive secondary metabolites (Weissman & Müller, 2010). To date more than 100 substances, many with promising antibiotic (Baumann et al., 2014; Surup et al., 2014), antiviral (Plaza et al., 2012), antifungal (Gerth, Bedorf, Irschik, Höfle, & Reichenbach, 1994), or anticancer activity (Gerth, Bedorf, Höfle, Irschik, & Reichenbach, 1996), could be isolated from myxobacteria and the limit has not been reached. Particularly in times of increasing antibiotic resistances the discovery and development of new antibiotics is of high importance. In the past, particularly new myxobacterial families and genera turned out to be reliable sources for new bioactive metabolites (Garcia, Stadler, Gemperlein, & Müller, 2015; Jansen, Mohr, Bernecker, Stadler, & Müller, 2014; Karwehl et al., 2015; Mohr, Garcia, Gerth, Irschik, & Müller, 2012; Plaza et al., 2012; Sood et al., 2014; Steinmetz et al., 2012). But based on cultivation, approximately only 1% of the naturally occurring bacterial diversity has been isolated and characterized so far (Muyzer, 1999). Most bacterial groups remain uncultured and uncharacterized because appropriate culture conditions remain to be found (Vaz‐Moreira, Silva, Manaia, & Nunes, 2008). These uncultured bacteria are an untapped source of new antibiotics (Lewis, 2013). To get access to new hitherto uncultivated groups, two main approaches may lead to success: Beside innovative isolation techniques like, for example, cultivation in diffusion chambers (Kaeberlein, Lewis, & Epstein, 2002) or on isolation chips (ichip) (Nichols et al., 2010), the intensive examination of neglected/extreme habitats is promising. But which habitat harbors (new) unknown myxobacteria? A prior inventory of the myxobacterial diversity of a so far uninvestigated habitat is useful before time‐ and cost‐consuming extensive large‐scale cultivation approaches are considered with samples which possibly lack myxobacteria. Until now, extreme habitats like humic acid rich pine forests or acidic moors are neglected habitats with regard to the isolation of myxobacteria. Less is known about the myxobacterial diversity in these habitats and most studies are several decades old. Therefore, we investigated high moor and fen samples due to their myxobacterial diversity with cultivation‐independent methods, based on PCR amplification and sequencing of 16S rRNA genes, as well as with cultivation. As described in our previous study (Mohr, Stechling, Wink, Wilharm, & Stadler, 2016), a semi‐selective primer pair from the literature (Wu, Jiang, Li, & Li, 2005), with forward primer specific for *Sorangiineae*/*Nannocystineae* and *Cystobacterineae*, respectively, and eubacterial reverse primer was used for PCR. The sequences were compared to the 16S rRNA genes of the received cultures and to sequences of a public database (NCBI). Furthermore, we adapted the standard cultivation methods for the isolation of myxobacteria (Shimkets, Dworkin, & Reichenbach, 2006) to environmental conditions of moors and fens. Finally, we integrated the sequences (clones and cultures) into a phylogenetic tree containing all myxobacterial type strains to provide new insight into the uncommon habitat moor.

## 2 Experimental Procedures

### 2.1 Soil sample collection

The samples were taken in July 2014 from high moor and fen of the Brockenfeld moor and from fen at the creek “Am Sandbeek” within the Harz region, Germany. The Brockenfeld moor is a high moor with fen‐like regions at the edge. The edge region is 875 m above sea level (51°47′12.52″ N; 10°34′6.19″ E). A total of four samples (three soil samples and one water sample) were collected from the fen‐like habitat of the Brockenfeld moor and are listed in Table S6 as samples A1–A4. The second characteristic sampling site was the central high moor (Brockenfeld), located 877 m above sea level (51°47′13.23″ N; 10°34′13.48″ E). All in all 29 samples (17 from soil, 8 feces, and 4 water samples) were collected from Brockenfeld high moor (B1–B29; Table S6). The dominant flora at the Brockenfeld high moor is typical *Sphagnum* moss *Trichophorum cespitosum*,* Melampyrum pratense*,* Vaccinium* spp., and *Eriophorum vaginatum*. The Brockenfeld moor is crossed by several drainage channels. The third sampling site was a fen at the creek “Sandbeek”. This habitat has swampy moor character, and is located 778 m above sea level (51°46′14.2″ N; 10°35′32.17″ E). Four soil samples and one water sample were collected here (C1–C5; Table S6). Dominant fauna is composed of *Lycopodium* sp., *Drosera* sp., *Carex echinata*,* Carex rostrata, Eriophorum vaginatum,* and several mosses. The fen samples were much more humid than the high moor samples, which were slightly damp to dry. Numerous puddles are characteristic for this habitat. The samples were collected from the upper layer to a depth of a few centimeters in 50 ml Falcon tubes (fill level: 2/3), each tube was mixed and the samples were directly used for DNA extraction and cultivation. PH was determined with commercial pH stripes (Roth). After first cultivation approaches, the samples were air dried and used for several months as raw material for further cultivation approaches.

### 2.2 Isolation of predators with bait bacteria isolated from the moor samples

Isolation of bait bacteria is described in detail in the supplement.

### 2.3 Isolation of myxobacteria from moor samples with adapted isolation procedures

Myxobacteria were isolated according to the standard methods described at Shimkets et al. (2006), but with some modifications: pH was adjusted to 5.0 or 6.0, incubation at room temperature (instead of 30°C), offering *E. coli* DSM 1116 but also eight different moor‐bacteria as bait, and samples were used fresh and also after drying at room temperature. Water samples were treated as described in the supplement. Water‐agar (20 g agar/L) and Stan21‐agar were prepared as described at Shimkets et al. (2006), but with filtered moorwater (pH 5.33). The further steps were as described for the standard procedure. Each sample was spotted on water agar/Stan 21 at least for 20 times. All sample approaches were incubated at room temperature for several weeks and examined for myxobacterial growth or fruiting body production every few days using a dissecting microscope. Fruiting bodies or material from swarm edges visible on the raw culture plates were transferred to new water agar plates with *E. coli* and finally to CY or VY/2 agar, respectively (Reichenbach & Dworkin, 1992). Pure cultures were transferred from the agar plates to 20 ml CY/H‐medium [per liter: 1.0 g defatted soy flour, 1.0 g glucose, 4.0 g starch (Cerestar), 1.5 g yeast extract, 1.5 g casitone, 1.0 g CaCl2 • 2H2O, 0.5 g MgSO4 • 7H2O, 0.008 g iron EDTA, 11.8 g HEPES, pH 7.3]. After a few days, cultures were scaled up to 100 ml CY/H‐medium. Portions of 1.5 ml of well grown cultures were conserved at −80°C.

### 2.4 DNA extraction from cultures

DNA of pure cultures was extracted with the Spin Plant Mini Kit (Invisorb) according to the provider's instruction.

### 2.5 PCR conditions for amplification of 16S rRNA genes from cultures and sequence analyses

PCR reaction was conducted with eubacterial primers F27/R1525 and described in detail in the supplement. 16S rRNA genes of all cultures were sequenced using primer F27 and F518. From three representative cultures (B2, B19, B29‐2), almost full‐length 16S rRNA genes were sequenced using additionally primer F1100, R1100, and R1525. The sequences of all cultures have been deposited at GenBank under accession numbers KX810169–KX810192.

### 2.6 DNA extraction from soil

For clone bank analyses, two samples from central high moor (B4, B9) and two from central fen (C1 and C2) were used. Total DNA was extracted using the PowerSoil^®^DNA Isolation Kit from MoBio Laboratories following the manufacturer's instruction. Triplicates of the four samples (25 mg each) were extracted in parallel and the quality of genomic DNA was checked by agarose gel (0.8%). High‐quality DNA was combined and used for PCR.

### 2.7 Establishment of clone bank analyses

Direct PCR with genomic soil DNA and semi selective primer for myxobacteria failed. Thereupon, a nested PCR‐approach was used with universal bacterial primer F27/R1525 in triplicates (Lane, 1991; Stackebrandt, Liesack, & Goebel, 1993). The PCR conditions were as described for the cultures. Triplicates were combined and diluted 1:50 with water. For the myxobacteria‐specific clone bank analyses, two semi‐specific primer pairs were chosen from the literature: the universal reverse primer R1492 and the myxobacterial‐specific forward primer FW5 (Wu et al., 2005) for *Sorangiineae*/*Nannocystineae*, resulting in an amplicon of 963 bp and FW2 (Wu et al., 2005) for *Cystobacterineae* amplifying 1063 bp. PCR products were checked on agarose gel and purified using the NucleoSpin Gel and PCR clean up Kit (Macherey Nagel). Primers used in this study are listed in Table S7. Establishment of clone banks is also described at Mohr et al. (2016). Four hundred clone sequences of the expected length were chosen (50 per library) for sequencing using forward primer FW2 or FW5, respectively. Only sequences of high quality were further analyzed. Clone sequence lengths ranged between 399 bp and 900 bp. Sequences of clones were deposited in GenBank under accession numbers KU158476 to KU158753.

### 2.8 Data analysis

The 16S rRNA gene sequences acquired via clone bank and from the isolates were checked for quality using the program BioEdit (free available). The sequences from the cultures were assembled into consensus sequences. The single sequences from clones as well as the consensus sequences from cultures were compared with the NCBI database entries. Closely related myxobacterial sequences as well as sequences of the 56 myxobacterial type strains were imported into the ARB database (http://www.arb-home.de; Ludwig et al., 2004) and aligned together with the sequences acquired in this study. A distance matrix tree was constructed with 16S rRNA using the Neighbour‐Joining method (Saitou & Nei, 1987) and Jukes Cantor correction (Jukes & Cantor, 1969). The topology of the phylogenetic tree was built by bootstrap analysis of 1,000 operations. Sequences sharing ≥99% similarity, calculated with the similarity matrix tool in ARB, were grouped into operational taxonomic units (OTUs). For phylogenetic analyses, the 16S rRNA gene of three cultivated representatives was almost fully sequenced. Table S8 shows the type strains and their corresponding 16S rRNA gene accession numbers used for the construction of the phylogenetic tree. Single sequences or sequences of clones from OTUs which did not include type strain sequences were blasted in GenBank to figure out the next cultivated relative. In all cases, the next cultivated relative belongs to the *Myxococcales*. So, we are sure that all our clone sequences included in this study are of myxobacterial origin.

## 3 Results and Discussion

### 3.1 Myxobacterial diversity in high moor and fen detected with cultivation independent clone bank analyses

From a total of 38 collected samples which were all used for cultivation approaches, cost‐intensive clone banks were established from four representative sampling sites, two from high moor (B4, B9) and two from fen (C1, C2). 16S rRNA genes were amplified in a first PCR with eubacterial primers (F27/R1525), whereby total genomic soil DNA was used as template. Subsequently, these PCR products were used as template in a second, semi‐selective nested PCR with selective forward primer for *Cystobacterineae* (FW2), *Sorangiineae*/*Nannocystineae* (FW5) and eubacterial reverse primer (R1492). In a previous study, this combination led to the best results and highest yields of PCR‐products (Mohr et al., 2016). Eight clone banks have been established (four samples, two primer‐combinations FW2/R1492 and FW5/R1492 each). PCR products were ligated in plasmids, cloned in *Escherichia coli*, and sequenced. A total of 367 from 400 clone sequences showed PCR products of the expected length and were sequenced. After database comparison (NCBI) and chimera check, 70 non‐myxobacterial sequences (mainly acidobacteria) and 19 putative chimeras were excluded from further analyses. From the remaining 278 sequences, 173 clones emerged from high moor and 105 from fen (Table S1). The forward primer specificity within the *Myxococcales* was perfect: all clones which grouped into the *Cystobacterineae* originate from PCR products amplified with forward primer FW2 (specific for *Cystobacterineae*), all clones which grouped into the *Sorangiineae* were from PCR products amplified with forward primer FW5 (specific for *Sorangiineae*/*Nannocystineae*). The clones distribute among 23 operational taxonomic units (OTUs) based on 99% sequence similarity as shown in Table S2. Clones from other studies of acidic soils which show ≥99% similarity to clones of our study were also integrated in the OTUs (column “NCBI” in Table S2). The OTU‐affiliation of every single clone sequence is shown in Table S3. From 278 clones 214 grouped into 19 *Sorangiineae*‐OTUs and four *Cystobacterineae*‐OTUs, which contain at least two sequences. Phylogenetic analyses revealed 111 clones as members of *Cystobacterineae* and 167 as *Sorangiineae*. However, no clones grouped into the *Nannocystineae*.

**Figure 1:**
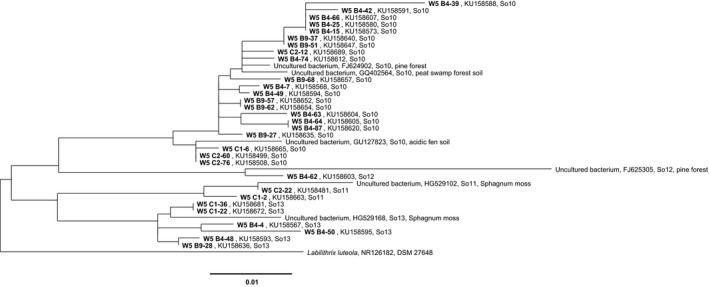
Section of a distance matrix tree based on 16S rRNA‐gene sequences of all 56 myxobacterial type strains and clone sequences from the present study as well as sequences from other cultivation‐independent studies of acidic soils. For a better clarity, only a section of the whole tree is shown. Sequences from OTUs So10–So13 are shown. W5: forward primer specific for *Sorangiineae*/*Nannocystineae*. C1 and C2: sampling site fen Am Sandbeek, soil; B4 and B9: Brockenfeld central high moor, soil. The section shows part of the *Sorangiineae* subfamily. Bar, 0.01 substitutions per nucleotide position. The full tree is shown as Figure S2

In‐silico analyses of primer FW5, specific for *Sorangiineae*/*Nannocystineae*, revealed 2–3 mismatches for the eight type‐strains of *Nannocystineae*. Therefore, we also checked primer combination FW5‐R1492 with genomic DNA of five *Nannocystineae* type strains and got PCR products of very good quality and quantity for all tested strains, demonstrating that this combination is in principle suitable for the detection of *Nannocystineae* (Figure S1). Four out of six genera of the *Nannocystineae* suborder are described from marine or estuary environments, namely *Enhygromyxa*,* Haliangium*,* Plesiocystis*, and *Pseudenhygromyxa*. The remaining genera *Nannocystis* and *Kofleria* have not been mentioned in publication about myxobacteria from moors so far (Dawid, 1984; Hook, 1977; Rückert, 1979), so maybe moors are not a suitable habitat for members of the *Nannocystineae*. None of the 23 OTUs of our study contains sequences from cultures, neither from our nor from other studies. However, 11 OTUs contain sequences from other cultivation‐independent studies of mainly acidic soils, like wetland peat bog (Liu et al., 2014), boreal pine forest (unpublished), *Sphagnum* moss in Finland (Putkinen et al., 2014), acidic fen soil (unpublished), or tropical peat swamp forest soil (Kanokratana et al., 2011; Figure 1; column “NCBI” in Table S2). Sixty‐four sequences showed less than 99% similarity and were designated as “single”. Thirty‐two singles are from high moor and 32 from fen and they also group in equal parts to *Sorangiineae* and *Cystobacterineae*. Twenty‐three OTUs contain sequences from high moor, 20 contain those from fen. Eight OTUs are composed of sequences from both habitats (high moor and fen), eight OTUs exclusively contain sequences from high moor and seven exclusively from fen. However, these OTUs only contain 2–7 sequences (Table S2). Therefore, it can be assumed that the diversity of myxobacteria detected with this cultivation‐independent method is similar in high moor and fen. The largest OTUs are the *Cystobacterineae*‐OTU Cb2 containing 91 clones, the *Sorangiineae*‐OTU So3 with 47 clones, and So10 with 24 clones (Figure S2). These OTUs contain clones from all sites and also from other studies, so it can be assumed that these myxobacteria are widespread in high moor and fen but resist various cultivation approaches. As none of the clone sequences is related to cultivated myxobacteria, moors are obviously a highly potent source for the isolation of unknown species, genera and even families of myxobacteria.

### 3.2 Bait bacteria isolated from moor samples

Eight non‐myxobacterial strains could be isolated from moor samples (Table S4) and were offered as bait instead of *E. coli* (DSM 1116) in order to attract predatory myxobacteria. From all plates where moor‐bacteria were offered as bait, myxobacteria could be isolated. However, no other species of myxobacteria than those attracted by *E. coli* (DSM1116), used as standard bait in our lab, could be detected.

### 3.3 Cultivation of myxobacteria from high moor and fen

Using enhanced standard cultivation methods more than 30 predatory myxobacterial strains could be isolated from 23 of 38 samples. Potential duplicates (strains isolated from the same sample showing same morphology) were excluded from further analyses. Finally, a total of 24 cultures, 17 from Brockenfeld high moor, 2 from Brockenfeld fen, and 5 from fen samples Am Sandbeek were included in this study (Table S5). Based on 16S rRNA gene analyses, the next related myxobacterial type strain of the 24 cultures is [*Melittangium lichenicola*] DSM 2275^T^ (99.2%–99.3%).

**Figure 2:**
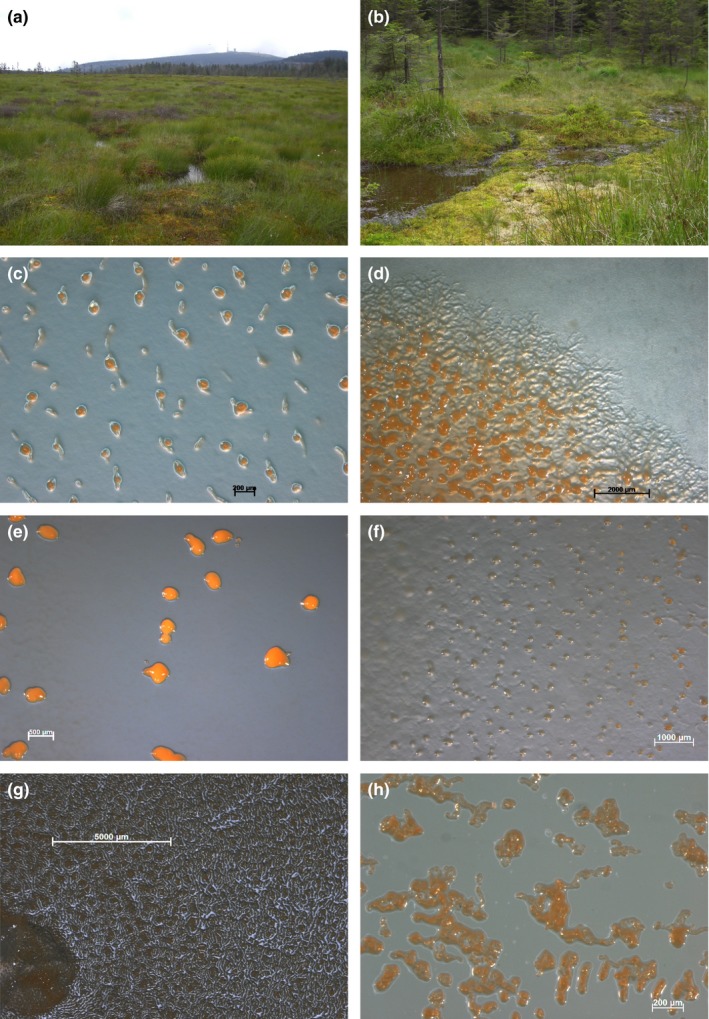
(a) Brockenfeld high moor; (b) Fen Am Sandbeek; (c–h) Photographs showing the morphology of isolated strains from moor. Phase‐contrast photomicrographs of (c) B2 fruiting bodies on CY agar; (d) fruiting bodies and swarm on P‐agar (e) B19 fruiting bodies on VY/2 agar (f) B29‐2 fruiting bodies on VY/2 agar (g) *Corallococcus exiguus* (DSM 14696^T^) fruiting bodies and swarm on CY‐agar; (h) *C. coralloides* (DSM 2259^T^) fruiting bodies on water agar with *E. coli* bait

**Figure 3:**
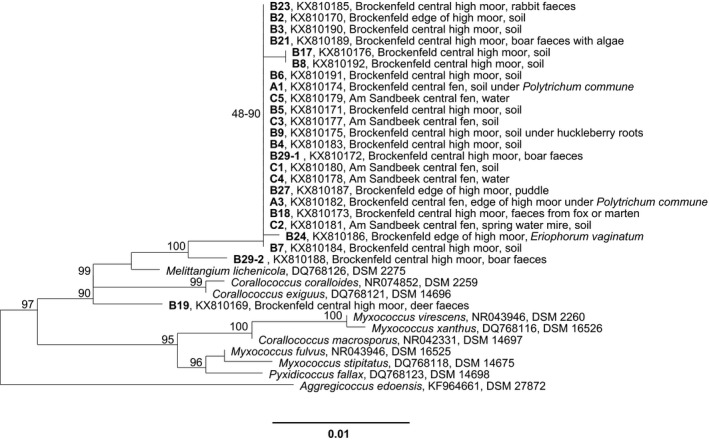
Section of a distance matrix tree based on 16S rRNA‐gene sequences of myxobacterial type strains and cultures isolated from moor. Bootstrap values >70% are shown. The section presents *Myxococcaceae*‐family. Bar, 0.01 substitutions per nucleotide position

However, due to swarm appearance, myxospore morphology and 16S rRNA gene sequence data Lang and Spröer (2008) proposed that this currently valid type strain of [*M*. *lichenicola*] DSM 2275^T^ (Acc.‐no: DQ768126) is clearly a member of the *Myxococcus*–*Corallococcus* clade and should be replaced by *M. lichenicola* DSM 14877 (AM930267). To this strain, the moor cultures show less than 96.4% similarity on basis of the 16S rRNA‐gene. Therefore, all moor cultures belong to *Myxococcaceae*‐family and show highest similarity (98.6%–99.0%) to the next valid type strain *Corallococcus exiguus* (DSM 14696^T^; AJ811598). Twenty‐two strains, isolated from both sampling sites, high moor and fen, show almost 100% sequence similarity to each other, although the sampling material was diverse (Table S6) and the sampling sites were meters or even kilometers away from each other (like Brockenfeld site and site Am Sandbeek). The 16S gene of one representative of this bulk, namely B2 (Figure 2), was almost fully sequenced (1483 bp). Two strains, B19 and B29‐2 (Figure 2) differ from this bulk (99.0%–99.2% similarity) and from each other (98.9% similarity). From these strains, 1,426 bp and 1,444 bp were sequenced (Figure 3). Interestingly, the type strains of the genus *Corallococcus*, namely *C. coralloides* (DSM 2259^T^; AJ811588) and *C. exiguus* (DSM 14696^T^; DQ768121), show almost high identity (99.8% sequence similarity) to each other. However, due to morphological differences, among other features, these strains have been described as representatives of independent species, although in general about 97.0% sequence similarity (Stackebrandt & Goebel, 1994) or 98.2%–99.0% (Meier‐Kolthoff, Göker, Spröer, & Klenk, 2013) are the common threshold for the description of new bacterial species. Cultures B2 (representative of the bulk cultures), B19 and B29‐2 show morphological and physiological differences from each other as well as from the type strains (Figure 2).

Further analyses will show whether these differences are sufficient to describe new *Corallococcus* species. Members of *Corallococcus* were the only and dominant species cultivable from almost all samples of different origin (soil, water, plants, dead wood, feces) from high moor and fen, and these strains seem to be either very well adapted to the acidic habitat moor and/or cope best with the isolation procedure.

*Sorangium cellulosum* could be observed on a raw culture plate (site C2, fen Am Sandbeek), but could not be purified (data not shown). Only a few publications about cultivable myxobacteria from acidic soils exist and most of them were conducted decades ago. Within our study, the diversity of cultivated myxobacteria from moor samples is very low. However, this is in accordance with other investigations like those by Hook (1977), who studied myxobacterial fruiting bodies which developed on the soil samples and distinguished 10 species on samples from moor water, but only five from adjacent soils of an alkaline bog, *Myxococcus* spp., *C. coralloides*,* Archangium gephyra*,* Melittangium lichenicola*, and *Sorangium cellulosum* (from 600 numbers of inoculation!). He also found *Corallococcus coralloides*, (formerly *Myxococcus coralloides*) to be dominant in terrestrial samples. Rückert (1979) also described *C. coralloides* as the predominant species in soils of pH 4.1–4.9 and as dominant as *M. fulvus* in soils of pH 3.0–3.5. In alpine acidic soils, *C. coralloides* was the third‐dominant species after two *Myxococcus* species. Rückert also noted that the overall myxobacterial diversity in acidic soils (pH 3.5–4.9) was less than in slightly acidic or neutral environments (pH 5.0–7.8). However, the pH of moor samples analyzed in our study is between 4.0 and 7.0 and therefore comparable to those from the other studies. Later, in 1984, Dawid isolated *Myxococcus xanthus*,* M. virescens* and *Polyangium* sp., but no cellulolytic species from undisturbed *Sphagnum* bogs of the Hohen Venn, Belgium.

A surprisingly high diversity of unknown myxobacteria in various samples of high moor and fen, detected exclusively with clone banks, faces the limited set of cultivated species (Figure S2). When cultivation‐independent analyses became more and more popular, also acidic habitats were investigated in more detail and compared in terms of cultivable and uncultivable bacteria. One of this studies dealt with the phylogenetic analysis and in situ identification of bacteria community composition in an acidic *Sphagnum* peat bog by 16S rRNA clone libraries, fluorescence in situ hybridization (FISH) and cultivation (Dedysh, Pankratov, Belova, Kulichevskaya, & Liesack, 2005). Dedysh and colleagues also detected a large discrepancy because among 84 environmental 16S rRNA gene clones, a set of only 16 cloned sequences was closely related (>95% similarity) to taxonomically described organisms and only one clone was myxobacteria‐related and showed distant relationship (97.2%) to the only known facultative anaerobic myxobacterium *Anaeromyxobacter dehalogenans*.

Therefore, isolation efforts under anaerobic conditions maybe a good chance of being successful with samples from deeper regions of moors. However, although if challenging isolation of anaerobic myxobacteria would be successful, subsequent screening for secondary metabolites and large scale production of new substances is only feasible with the appropriate equipment.

The detection‐cultivation anomaly was described for many bacterial groups including the *Myxococcales*: Wu et al. (2005); Jiang, Wu, Zhao, and Li (2007); Jiang et al. (2010), and Mohr et al. (2016) compared cultivable myxobacteria in a soil niche (Wu), campus garden soil (Jiang), deep‐sea sediments and hydrothermal vent (Jiang), Kiritimati sand and German compost (Mohr) to those detectable with cultivation‐independent methods. All authors could only isolate a small fraction of myxobacteria compared to the rich diversity detected by cultivation‐independent approaches.

Therefore, they suggest that myxobacteria in nature are much more diverse than assumed. Li et al. (2012) investigated the myxobacterial community in freshwater lake mud using high‐throughput 454 pyrosequencing and using myxobacteria‐enriched libraries with *Cystobacterineae*‐ and *Sorangiineae*‐specific primer pairs, respectively. They found out that the unclassified *Myxococcales* in the lake mud comprise a large portion of the population and exhibit high species diversity. Brinkhoff et al. (2012) studied the biogeography and phylogenetic diversity of a cluster of marine myxobacteria (MMC) which was exclusively detected by molecular, cultivation‐independent methods in marine sediments from the North and Mediterranean Sea, Pacific, Atlantic and Indian Ocean, but not in freshwater habitats. The putative high number of hitherto uncultivated myxobacteria may hold great potential for the discovery of new interesting bioactive substances. New isolation procedures as well as the investigation of uncommon or extreme habitats are promising. Using anaerobic cultivation approaches, for example, can open access to new taxa: Sanford, Cole, and Tiedje (2002) isolated the first taxon within the order *Myxococcales* capable of facultative anaerobic growth, *Anaeromyxobacter dehalogenans*, and it is highly unlikely that there are no further species, genera or families which are able to grow under facultative or even strictly anaerobic conditions. In addition, Iizuka and co‐workers were very successful in isolating and describing new myxobacterial genera and species from saline coastal and marine samples like *Haliangium ochraceum* and *H. tepidum* (Fudou, Jojima, Iizuka, & Yamanaka, 2002), Enhygromyxa *salina* Iizuka, Jojima, Fudou, Tokura, et al., 2003), *Plesiocystis pacifica* (Iizuka, Jojima, Fudou, Hiraishi, et al., 2003; and *Pseudenhygromyxa salsuginis* (Iizuka et al., 2013).

Soil samples B4, B9, C1, and C2 were used for clone bank analyses. From all of these samples, *Corallococcus*‐strains could be isolated. However, it is difficult to explain that no clone was related to cultures of our study. In silico analyses revealed that forward primer FW2, specific for *Cystobacterineae*, matches 100% with sequences of the three representative *Corallococcus*‐cultures of our study (B2, B19, B29‐2). Reverse primer R1492 also matches 100% on B2t‐1 and B29‐2 (sequence of MxB19t‐1 is too short for comparison). Therefore, we tested DNA of these strains in PCR (FW2‐R1492) and got PCR products of high quality and quantity (Figure S1). In our previous study (Mohr et al., 2016), several clones and one culture grouped into the *C. coralloides*/*C. exiguus*‐OTU (although clone banks were established with primer‐combination FW2 and eubacterial R1525), showing that the isolated *Corallococcus*‐species are in principle detectable with cultivation‐independent clone bank analyses. Some clone sequences from the previous study show more than 99% similarity to cultures of the present study (data not shown). However, during the work on our myxobacterial strain collection at HZI (more than 8,000 strains), we extracted DNA in a serial process from all investigated strains and found most *Corallococcus*‐strains to be highly resistant to various DNA extraction methods, although DNA‐extraction is performed with fresh, well grown pure cultures. Maybe *Corallococcus*‐strains were only present in form of myxospores, from which DNA is hardly extractable. Or the vegetative cells/myxospores of *Corallococcus* spp. were strongly under‐represented and/or therefore resist the total genomic DNA‐extraction procedure and subsequent PCR amplification in this study. Nevertheless, our results show that this habitat hides a high diversity of hitherto unidentified and therefore highly interesting myxobacteria with respect to their possible potential as producers of new secondary metabolites.

## Conflict of Interest

None declared.
