# Supplementary material for: Myxobacteria in high moor and fen: An astonishing diversity in a neglected extreme habitat
Source: Microbiologyopen. 2017 Apr 11;6(4):e00464. doi: 10.1002/mbo3.464 (PMC5552953; doi:10.1002/mbo3.464)
Supplement: Supplementary file 1 [file MBO3-6-na-s001.pdf]

Myxobacteria in high moor and fen: an astonishing diversity in a neglected extreme Habitat

Kathrin Irene Mohr,[1] Tanja Zindler,[2,3] Joachim Wink,[2] Elke Wilharm[3], and Marc Stadler [1,*]

[1]Microbial Drugs, [2]Microbial Strain Collection, Helmholtz Centre for Infection Research, Inhoffenstrasse 7, D-38124 Braunschweig, Germany, [3]Department of Supply Engineering, Ostfalia, Salzdahlumer Straße 46/48 D-38302 Wolfenbüttel, Germany.

*For correspondence. E-mail marc.stadler@helmholtz-hzi.de; Tel. +49 531 6181 4240; Fax +49 6181 9499

Supplemental Figures

Figure S1: Agarose gel used for primercheck.

Figure S2: Distance matrix tree based on 16S rRNA-gene sequences of all myxobacterial type strains and clone sequences and representative cultures from the present study.

Supplemental Tables

Table S1: Origin of clones achieved in this study.

Table S2: Number and source of clones and affiliation to OTUs.

Table S3: Sequences from clones and cultures emerged from our study with GeneBank Accession numbers, sequence length, and OTU affiliation.

Table S4: Bait organisms isolated from moor samples.

Table S5: Myxobacteria isolated within this study.

Table S6: Description of samples collected within this study.

Table S7: Primers used in this study.

Table S8: Type-strains of myxobacterial species used for the construction of the phylogenetic core tree, representative cultures of each OTU and sequences with high similarity to sequences of this study.

Supplemental Tables

Table S1. Origin of clones achieved in this study with Cystobacterineae-specific forward primer W2 and Nannocystineae/Sorangiineae specific forward primer W5; **B4**/**B9**: soil from Brockenfeld high moor; **C1**: dry soil from fen Am Sandbeek; **C2**: swampy soil from fen Am Sandbeek

	clone sequences	
**high moor**		
W2 – B4	34	
W2 – B9	43	
W5 – B4	56	
W5 – B9	40	
total		173
**fen**		
W2 – C1	32	
W2 – C2	2	
W5 – C1	23	
W5 – C2	48	
total		105

Table S2. Number and source of clones and affiliation to OTUs. Cystobacterineae (Cb) and Sorangiineae (So). "Single" sequences show less than 99% similarity to the remaining sequences. **B4**/**B9**: soil from Brockenfeld high moor; **C1**: dry soil from fen Am Sandbeek; **C2**: swampy soil from fen Am Sandbeek. NCBI*: clones from other studies, mainly from acidic soils

**OTU**	**sampling sites**				**clones**		
	**B4**	**B9**	**C1**	**C2**	this study	NCBI*	total
**So1**		2			2		2
**So2**	1	1	1		3	1	4
**So3**	16	14	3	12	45	2	47
**So4**	2				2		2
**So5**	3				3		3
**So6**		3	1		4		4
**So7**			2		2		2
**So8**	1	1		2	4		4
**So9**			1	3	4		4
**So10**	11	6	1	3	21	3	24
**So11**			1	1	2	1	3
**So12**	1				1	1	2
**So13**	3	1	2		6	1	7
**So14**			2		2		2
**So15**				2	2		2
**So16**	1				1	1	2
**So17**	1			1	2		2
**So18**				1	1	1	2
**So19**			2		2	1	3
**Cb1**	3	2			5		5
**Cb2**	29	29	31	1	90	1	91
**Cb3**		3			3		3
**Cb4**		7			7	1	8
**singles**	32		32		64		
**total**					**278**		292

Table S3. Sequences from clones emerged from our study with GeneBank Accession numbers, sequence length, and OTU affiliation

clone	Acc-no.	bp	OTU
**W5C2-17**	KU158476	730	So2
**W5C2-18**	KU158477	658	So3
**W5C2-19**	KU158478	799	So3
**W5C2-16**	KU158479	463	So18
**W5C2-27**	KU158480	645	So9
**W5C2-22**	KU158481	421	So11
**W5C2-21**	KU158482	488	single
**W5C2-35**	KU158483	608	So17
**W5C2-30**	KU158484	862	single
**W5C2-33**	KU158485	484	So3
**W5C2-34**	KU158486	708	So3
**W5C2-28**	KU158487	859	single
**W5C2-42**	KU158488	717	single
**W5C2-37**	KU158489	748	single
**W5C2-38**	KU158490	781	single
**W5C2-46**	KU158491	699	single
**W5C2-47**	KU158492	672	single
**W5C2-48**	KU158493	806	So9
**W5C2-43**	KU158494	457	single
**W5C2-56**	KU158495	712	So3
**W5C2-53**	KU158496	780	So3
**W5C2-54**	KU158497	458	single
**W5C2-55**	KU158498	621	So3
**W5C2-60**	KU158499	738	So10
**W5C2-62**	KU158500	565	So3
**W5C2-57**	KU158501	771	single
**W5C2-71**	KU158502	665	single
**W5C2-67**	KU158503	705	So9
**W5C2-66**	KU158504	760	So8
**W5C2-77**	KU158505	561	So3
**W5C2-74**	KU158506	429	So3
**W5C2-75**	KU158507	706	single
**W5C2-76**	KU158508	812	So10
**W5C2-84**	KU158509	701	single
**W5C2-79**	KU158510	747	single
**W5C2-86**	KU158511	827	single
**W5C2-87**	KU158512	780	So3
**W5C2-88**	KU158513	702	single
**W5C2-89**	KU158514	680	So3
**W5C2-85**	KU158515	791	single
**W5C2-94**	KU158516	810	So15
**W2B4-16**	KU158517	557	Cb2
**W2B4-21**	KU158518	540	Cb2
**W2B4-24**	KU158519	531	Cb2
**W2B4-30**	KU158520	654	Cb2
**W2B4-41**	KU158521	457	Cb2
**clone**	**Acc-no.**	**bp**	**OTU**
**W2B4-46**	KU158522	559	Cb2
**W2B4-47**	KU158523	710	Cb2
**W2B4-5**	KU158524	400	Cb2
**W2B4-51**	KU158525	594	Cb2
**W2B4-54**	KU158526	650	Cb2
**W2B4-57**	KU158527	736	Cb2
**W2B4-58**	KU158528	653	Cb1
**W2B4-59**	KU158529	649	Cb2
**W2B4-60**	KU158530	630	Cb1
**W2B4-64**	KU158531	660	Cb2
**W2B4-67**	KU158532	731	Cb2
**W2B4-68**	KU158533	713	Cb2
**W2B4-69**	KU158534	707	Cb2
**W2B4-7**	KU158535	556	Cb2
**W2B4-70**	KU158536	628	Cb2
**W2B4-73**	KU158537	548	single
**W2B4-74**	KU158538	629	Cb2
**W2B4-76**	KU158539	718	Cb2
**W2B4-81**	KU158540	713	Cb2
**W2B4-82**	KU158541	636	Cb2
**W2B4-83**	KU158542	594	Cb2
**W2B4-84**	KU158543	708	Cb2
**W2B4-86**	KU158544	548	Cb2
**W2B4-87**	KU158545	485	single
**W2B4-89**	KU158546	664	Cb2
**W2B4-90**	KU158547	714	Cb2
**W2B4-92**	KU158548	641	Cb2
**W2B4-94**	KU158549	554	Cb2
**W2B4-95**	KU158550	653	Cb1
**W2B9-1**	KU158551	461	Cb1
**W2B9-12**	KU158552	521	Cb2
**W2B9-16**	KU158553	589	Cb2
**W2B9-17**	KU158554	540	Cb2
**W2B9-18**	KU158555	507	Cb4
**W2B9-19**	KU158556	743	Cb2
**W2B9-2**	KU158557	561	single
**W2B9-3**	KU158558	557	Cb2
**W2B9-30**	KU158559	602	Cb2
**W2B9-33**	KU158560	768	Cb2
**W2B9-34**	KU158561	530	Cb2
**W2B9-39**	KU158562	712	Cb4
**W2B9-4**	KU158563	674	Cb2
**W2B9-41**	KU158564	399	Cb4
**W2B9-7**	KU158565	601	Cb2
**W5B4-3**	KU158566	642	So3
**W5B4-4**	KU158567	772	So13
**W5B4-7**	KU158568	826	So10
**W5B4-10**	KU158569	790	single
**W5B4-11**	KU158570	794	So3
**W5B4-12**	KU158571	779	single
**W5B4-14**	KU158572	772	single
**W5B4-15**	KU158573	523	So10
**clone**	**Acc-no.**	**bp**	

**W5B4-16**	KU158574	774	single
**W5B4-17**	KU158575	779	So3
**W5B4-18**	KU158576	762	So5
**W5B4-20**	KU158577	751	single
**W5B4-21**	KU158578	777	single
**W5B4-22**	KU158579	652	So5
**W5B4-25**	KU158580	766	So10
**W5B4-29**	KU158581	804	So4
**W5B4-30**	KU158582	765	single
**W5B4-32**	KU158583	564	single
**W5B4-33**	KU158584	777	single
**W5B4-34**	KU158585	723	So3
**W5B4-35**	KU158586	590	So17
**W5B4-36**	KU158587	750	single
**W5B4-39**	KU158588	769	So10
**W5B4-40**	KU158589	765	So3
**W5B4-41**	KU158590	744	single
**W5B4-42**	KU158591	767	So10
**W5B4-46**	KU158592	650	So3
**W5B4-48**	KU158593	777	So13
**W5B4-49**	KU158594	812	So10
**W5B4-50**	KU158595	755	So13
**W5B4-51**	KU158596	837	So2
**W5B4-53**	KU158597	642	So3
**W5B4-54**	KU158598	718	So3
**W5B4-55**	KU158599	682	So4
**W5B4-59**	KU158600	759	So3
**W5B4-60**	KU158601	719	single
**W5B4-61**	KU158602	801	So3
**W5B4-62**	KU158603	660	So12
**W5B4-63**	KU158604	720	So10
**W5B4-64**	KU158605	705	So10
**W5B4-65**	KU158606	744	single
**W5B4-66**	KU158607	766	So10
**W5B4-67**	KU158608	801	So5
**W5B4-71**	KU158609	751	So3
**W5B4-72**	KU158610	702	So3
**W5B4-73**	KU158611	689	So3
**W5B4-74**	KU158612	764	So10
**W5B4-75**	KU158613	721	single
**W5B4-76**	KU158614	795	single
**W5B4-77**	KU158615	768	So3
**W5B4-78**	KU158616	668	single
**W5B4-80**	KU158617	706	single
**W5B4-85**	KU158618	766	So8
**W5B4-86**	KU158619	773	So3
**W5B4-87**	KU158620	643	So10
**W5B4-88**	KU158621	645	So3
**W5B9-2**	KU158622	817	So3
**W5B9-3**	KU158623	648	So3
**W5B9-5**	KU158624	731	single
**W5B9-8**	KU158625	692	So3
**clone**	**Acc-no.**	**bp**	

**W5B9-10**	KU158626	657	So2
**W5B9-11**	KU158627	627	single
**W5B9-12**	KU158628	659	So3
**W5B9-13**	KU158629	753	So3
**W5B9-14**	KU158630	706	So8
**W5B9-20**	KU158631	689	So3
**W5B9-24**	KU158632	619	So3
**W5B9-25**	KU158633	659	So1
**W5B9-26**	KU158634	643	So1
**W5B9-27**	KU158635	707	So10
**W5B9-28**	KU158636	808	So13
**W5B9-29**	KU158637	516	single
**W5B9-32**	KU158638	697	single
**W5B9-33**	KU158639	646	single
**W5B9-37**	KU158640	792	So10
**W5B9-38**	KU158641	799	single
**W5B9-40**	KU158642	766	So3
**W5B9-41**	KU158643	809	So3
**W5B9-44**	KU158644	830	single
**W5B9-46**	KU158645	832	So3
**W5B9-48**	KU158646	802	single
**W5B9-51**	KU158647	736	So10
**W5B9-52**	KU158648	800	So3
**W5B9-53**	KU158649	801	So3
**W5B9-55**	KU158650	784	So6
**W5B9-56**	KU158651	612	single
**W5B9-57**	KU158652	697	So10
**W5B9-61**	KU158653	832	So3
**W5B9-62**	KU158654	771	So10
**W5B9-63**	KU158655	743	So6
**W5B9-67**	KU158656	658	single
**W5B9-68**	KU158657	493	So10
**W5B9-72**	KU158658	543	So3
**W5B9-76**	KU158659	499	So16
**W5B9-77**	KU158660	728	single
**W5B9-84**	KU158661	738	So6
**W5C1-1**	KU158662	791	So7
**W5C1-2**	KU158663	763	So11
**W5C1-5**	KU158664	836	So14
**W5C1-6**	KU158665	736	So10
**W5C1-9**	KU158666	769	So14
**W5C1-11**	KU158667	744	So9
**W5C1-12**	KU158668	541	single
**W5C1-13**	KU158669	716	So7
**W5C1-15**	KU158670	700	single
**W5C1-17**	KU158671	763	single
**W5C1-22**	KU158672	644	So13
**W5C1-25**	KU158673	762	single
**W5C1-26**	KU158674	716	So3
**W5C1-28**	KU158675	637	single
**W5C1-30**	KU158676	648	So3
**W5C1-32**	KU158677	710	single
**clone**	**Acc-no.**	**bp**	**OTU**

**W5C1-33**	KU158678	634	So19
**W5C1-34**	KU158679	783	single
**W5C1-35**	KU158680	764	So19
**W5C1-36**	KU158681	736	So13
**W5C1-39**	KU158682	725	single
**W5C1-41**	KU158683	782	So3
**W5C1-42**	KU158684	773	So6
**W5C2-5**	KU158685	583	single
**W5C2-6**	KU158686	663	single
**W5C2-8**	KU158687	775	single
**W5C2-11**	KU158688	646	So8
**W5C2-12**	KU158689	777	So10
**W5C2-13**	KU158690	695	single
**W5C2-14**	KU158691	638	So15
**W2B9-42**	KU158692	681	Cb2
**W2B9-43**	KU158693	723	Cb2
**W2B9-44**	KU158694	737	Cb2
**W2B9-45**	KU158695	764	Cb2
**W2B9-46**	KU158696	739	Cb2
**W2B9-47**	KU158697	710	Cb2
**W2B9-49**	KU158698	702	Cb2
**W2B9-52**	KU158699	691	Cb4
**W2B9-54**	KU158700	686	Cb2
**W2B9-55**	KU158701	650	Cb2
**W2B9-59**	KU158702	671	Cb2
**W2B9-61**	KU158703	705	Cb2
**W2B9-62**	KU158704	712	Cb2
**W2B9-67**	KU158705	780	single
**W2B9-68**	KU158706	640	Cb2
**W2B9-70**	KU158707	523	Cb3
**W2B9-74**	KU158708	586	Cb4
**W2B9-79**	KU158709	685	Cb2
**W2B9-82**	KU158710	731	Cb2
**W2B9-83**	KU158711	705	Cb2
**W2B9-86**	KU158712	686	Cb2
**W2B9-87**	KU158713	723	Cb4
**W2B9-88**	KU158714	671	Cb3
**W2B9-90**	KU158715	755	Cb1
**W2B9-91**	KU158716	719	Cb3
**W2B9-93**	KU158717	481	Cb2
**W2B9-94**	KU158718	569	Cb4
**W2B9-95**	KU158719	730	Cb2
**W2C1-10**	KU158720	414	Cb2
**W2C1-11**	KU158721	634	Cb2
**W2C1-12**	KU158722	675	Cb2
**W2C1-14**	KU158723	733	Cb2
**W2C1-16**	KU158724	723	Cb2
**W2C1-2**	KU158725	687	Cb2
**W2C1-20**	KU158726	596	Cb2
**W2C1-21**	KU158727	622	Cb2
**W2C1-23**	KU158728	653	Cb2
**W2C1-26**	KU158729	703	Cb2
**clone**	**Acc-no.**	**bp**	**OTU**

**W2C1-28**	KU158730	699	Cb2
**W2C1-31**	KU158731	536	Cb2
**W2C1-33**	KU158732	552	Cb2
**W2C1-34**	KU158733	635	Cb2
**W2C1-40**	KU158734	698	Cb2
**W2C1-41**	KU158735	678	single
**W2C1-50**	KU158736	627	Cb2
**W2C1-53**	KU158737	636	Cb2
**W2C1-56**	KU158738	542	Cb2
**W2C1-57**	KU158739	656	Cb2
**W2C1-58**	KU158740	522	Cb2
**W2C1-59**	KU158741	585	Cb2
**W2C1-64**	KU158742	681	Cb2
**W2C1-68**	KU158743	643	Cb2
**W2C1-70**	KU158744	703	Cb2
**W2C1-74**	KU158745	556	Cb2
**W2C1-75**	KU158746	630	Cb2
**W2C1-78**	KU158747	687	Cb2
**W2C1-83**	KU158748	693	Cb2
**W2C1-85**	KU158749	811	Cb2
**W2C1-86**	KU158750	710	Cb2
**W2C1-90**	KU158751	826	Cb2
**W2C2-24**	KU158752	764	single
**W2C2-79**	KU158753	791	Cb2

Table S4: Bait organisms isolated from moor samples and identified on basis of 16S rRNA sequences. The next relative in the NCBI database, the accession number of the relative, the sequence similarity (%) and the sequence length (bp) of the isolates is indicated.

**sample**	next relative/Acc-No.	sequence similarity (%)	sequence length (bp)
**FO1**	*Variovorax paradoxus* / KP686135	100	1265
**FO2**	*Pseudomonas* sp. / KR025492	100	1288
**FO3**	*Paenibacillus odorifer* / KJ781900	99.9	1400
**FO4**	*Paenibacillus taichungensis*/ LN889997	100	1026
**FO5**	*Arthrobacter citreus* / FM955881	100	855
**FO6**	*Bacillus* sp. *	*	1403
**FO7**	*Lysinibacillus varians* / LN870303	100	1168
**FO8**	*Escherichia coli* / CP013029	100	923

*100 % to several species of *Bacillus*

Table S5. Myxobacterial cultures isolated from Brockenfeld fen (A), Brockenfeld high moor (B) and fen Am Sandbeek (C), sequence length (bp) and accession numbers.

OTU	Sequence length (bp)	Acc. No.
**Brockenfeld fen**		
**A1**	1045	KX810174
**A3**	1033	KX810182
**Brockenfeld high moor**		
**B2**	1483	KX810170
**B3**	1204	KX810190
**B4**	1035	KX810183
**B5**	1040	KX810171
**B6**	1122	KX810191
**B7**	815	KX810184
**B8**	1195	KX810192
**B9**	1038	KX810175
**B17**	1041	KX810176
**B18**	1031	KX810173
**B19**	1426	KX810169
**B21**	1198	KX810189
**B23**	1316	KX810185
**B24**	882	KX810186
**B27**	1034	KX810187
**B29-1**	1035	KX810172
**B29-2**	1444	KX810188
**Am Sandbeek fen**		
**C1**	1035	KX810180
**C2**	984	KX810181
**C3**	1039	KX810177
**C4**	1034	KX810178
**C5**	1043	KX810179

Table S6. Sample name, origin, site description and sample material of the 38 samples collected in high moor and fen for this study.

sample	origin	site description	material
**A1**	**Fen Brockenfeldmoor**	central fen, edge of high moor under *Polytrichum commune*	soil
**A2**		edge of high moor	soil
**A3**		central fen, edge of high moor under *Polytrichum commune*	soil
**A4**		edge of high moor	water
**B1**	**High moor Brockenfeldmoor**	edge of high moor, quagmire	soil
**B2**		edge of high moor, quagmire	soil
**B3**		central high moor under huckleberry roots	soil
**B4**		central high moor	soil
**B5**		central high moor	soil
**B6**		central high moor beside spruce	soil
**B7**		central high moor	soil
**B8**		central high moor	soil
**B9**		central high moor under huckleberry	soil
**B10**		central high moor upper trench side	soil
**B11**		central high moor middle trench side	soil
**B12**		central high moor bottom trench side	soil
**B13**		central high moor trench side waterfront	soil
**B14**		central high moor trench side waterfront 2	soil
**B15**		central high moor trench side water leak	soil
**B16**		central high moor dry drainage ditch	soil
**B17**		central high moor	soil
**B18**		central high moor	fox or marten faeces
**B19**		central high moor	deer faeces
**B20**		edge of high moor	dead wood spruce
**B21**		central high moor	boar faeces with algae
**B22**		central high moor	exobasidio
**B23**		central high moor	rabbit faeces
**B24**		edge of high moor	*Eriophorum vaginatum*
**B25**		edge of high moor, puddle	water
**B26**		central high moor, puddle	water
**B27**		edge of high moor, puddle	water
**B28**		edge of high moor trench side	water
**B29**		central high moor	boar faeces
**C1**	**Fen Am Sandbeek**	central fen, dry	soil
**C2**		central fen, spring water mire	soil
**C3**		central fen	soil
**C4**		central fen, dry	water
**C5**		central fen, water run off	water

Table S7. Primers used in this study

primer	specificity	position[a]	primer sequences (5'-3')	references
F27	UB	8-27	AGA GTT TGA TCC TGG CTC AG	Lane *et al*. 1991
F518	UB	518-537	CCA GCA GCC GCG GTA ATA CG	Lane *et al*. 1991
F1100	UB	1100-1114	YAA CGA GCG CAA CCC	Lane *et al*. 1991
R1100	UB	1114-1100	GGG TTG CGC TCG TTG	Lane *et al*. 1991
R1525	UB	1525-1542	AAG GAG GTG ATC CAG CCG CA	Stackebrandt *et al*. 1993
R1492	UB	1492-1507	TAC GGY TAC CTT GTT ACG ACT T	Weisburg *et al*. 1991
FW2	CS	427–444	GTA AAG CAC TTT CGA CCG	Wu *et al*. 2005
FW5	SN	529–550	GTA AGA CAG AGG GTG CAA ACG T	Wu *et al*. 2005
pUC M13F	pGem-T		CGC CAG GGT TTT CCC AGT CAC GAC	Promega
pUC M13R	pGem-T		TCA CAC AGG AAA CAG CTA TGA C	Promega

[a]Corresponds to the sequence number of 16S rRNA in *Escherichia coli* (Brosius *et al*., 1978). UB, universal bacteria; CS: Cystobacterineae-specific; SN: Sorangiineae/Nannocystineae-specific; F: forward; R: reverse.

Brosius, J., Palmer, M.L., Kennedy, P.J., and Noller, H.F. (1978) Complete nucleotide sequence of a 16S ribosomal RNA gene from *Escherichia coli*. *Proc Natl Acad Sci* USA, **75:** 4801-4805.

Weisburg, W.G., Barns, S.M., Pelletier, D.A., and Lane, D.J. (1991) 16S ribosomal DNA amplification for phylogenetic study. *J Bacteriol* 173: 697–703.

Table S8. All type-strains of myxobacterial species with DSM- and Accession number used for the construction of the phylogenetic core tree, representative cultures of each OTU and additional sequences with high similarity to sequences of this study

**Suborder**	Family	Genus	Species	DSM	Acc.-no.
**Cystobacterineae**	**Myxococcaceae**	*Aggregicoccus*	*edonensis*	27872	KF914661
		Corallococcus	*coralloides*	2259	NR074852
			exiguus	14696	DQ768121
			macrosporus	14697	NR042331
		Myxococcus	*fulvus*	16525	NR043946
			stipitatus	14675	DQ768118
			virescens	2260	NR043946
			xanthus	16526	DQ768116
		Pyxidicoccus	*fallax*	14698	DQ768123
	**Cystobacteraceae**	*Archangium*	*gephyra*	2261	DQ768106
			disciforme	52716	NR117460
		Cystobacter	*armeniaca*	14710	DQ768107
			badius	14723	DQ768108
			ferrugineus	14716	AJ233901
			fuscus	2262	DQ768109
			gracilis	14753	DQ768110
			miniatus	14712	DQ768111
			minus	14751	AJ233903
			velatus	14718	DQ768115
			violaceus	14727	DQ768114
		Hyalangium	*minutum*	14724	DQ768124
		Melittangium	*boletus*	14713	AJ233908
			lichenicola	2275	DQ768126
		Stigmatella	*aurantiaca*	17044	GU207882
			erecta	16858	AJ970180
			hybrida	14722	DQ768129
			armeniaca	14710	DQ768107
	**Vulgatibacteraceae**	*Vulgatibacter*	*incomptus*	27710	AB847448

Suborder	Family	Genus	Species	DSM	Acc.-no.
	**Anaeromyxobacteraceae**	*Anaeromyxobacter*	*dehalogenans*	21875	AF382396
**Sorangiineae**		*Minicystis*	*rosea*	24000	GU249616
		Aetherobacter	*fasciculatus*	24601	GU249609
			rufus	24628	GU249610
		Byssovorax	*cruenta*	14553	AJ833647
		Chondromyces	*apiculatus*	14605	AJ233938
			crocatus	14714	GU207874
			lanuginosus	14631	AJ233939
			pediculatus	14607	GU207875
			robustus	14608	AJ233942
		Jahnella	*thaxteri*	14626	NR117461
		Polyangium	*sorediatum*	14670	GU207880
			fumosum	14668	GU207879
			spumosum	14734	GU207881
		Racemicystis	*crocea*	100773	KT591707
			iranensis	103165	KX443485
		Sorangium	*cellulosum*	14627	NR116678
	**Sandaracinaceae**	*Sandaracinus*	*amylolyticus*	53668	HQ540311
	**Phaselicystidaceae**	*Phaselicystis*	*flava*	21295	EU545827
	**Labilitrichaceae**	*Labilithrix*	*luteola*	27648	NR126182
**Nannocystineae**	**Nannocystaceae**	*Enhygromyxa*	*salina*	15217	NR024807
		Nannocystis	*exedens*	71	M94279
			pusilla	14622	NR117463
		Plesiocystis	*pacifica*	14875	NR024795
		Pseudenhygromyxa	*salsuginis*	21377	AB600195
	**Kofleriaceae**	*Kofleria*	*flava*	14601	AJ233944
		Haliangium	*ochraceum*	14365	AB016470
			tepidum	14436	AB062751

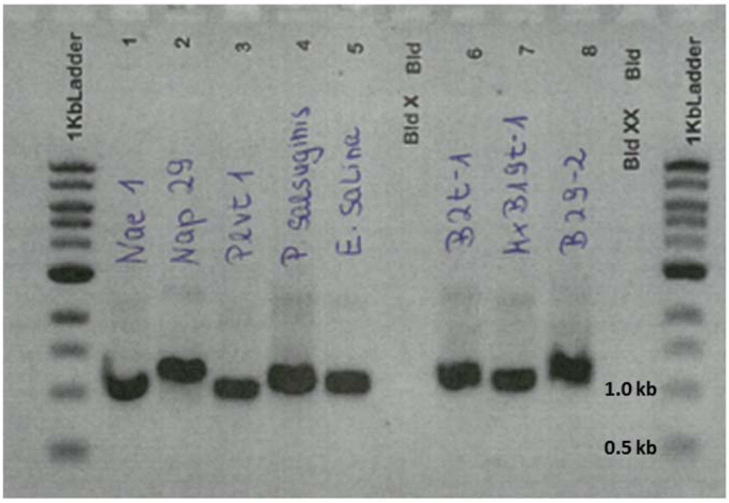

Primer W5F-1492R

1. *Nannocystis exedens*^{T}
2. *Nannocystis pusilla*^{T}
3. *Kofleria flava*^{T}
4. *Pseudenhygromyxa salsuginis*^{T}
5. *Enhygromyxa salina*^{T}

Primer W2F-1492R

1. B2
2. B19
3. B29-2

Figure S1. Primercheck

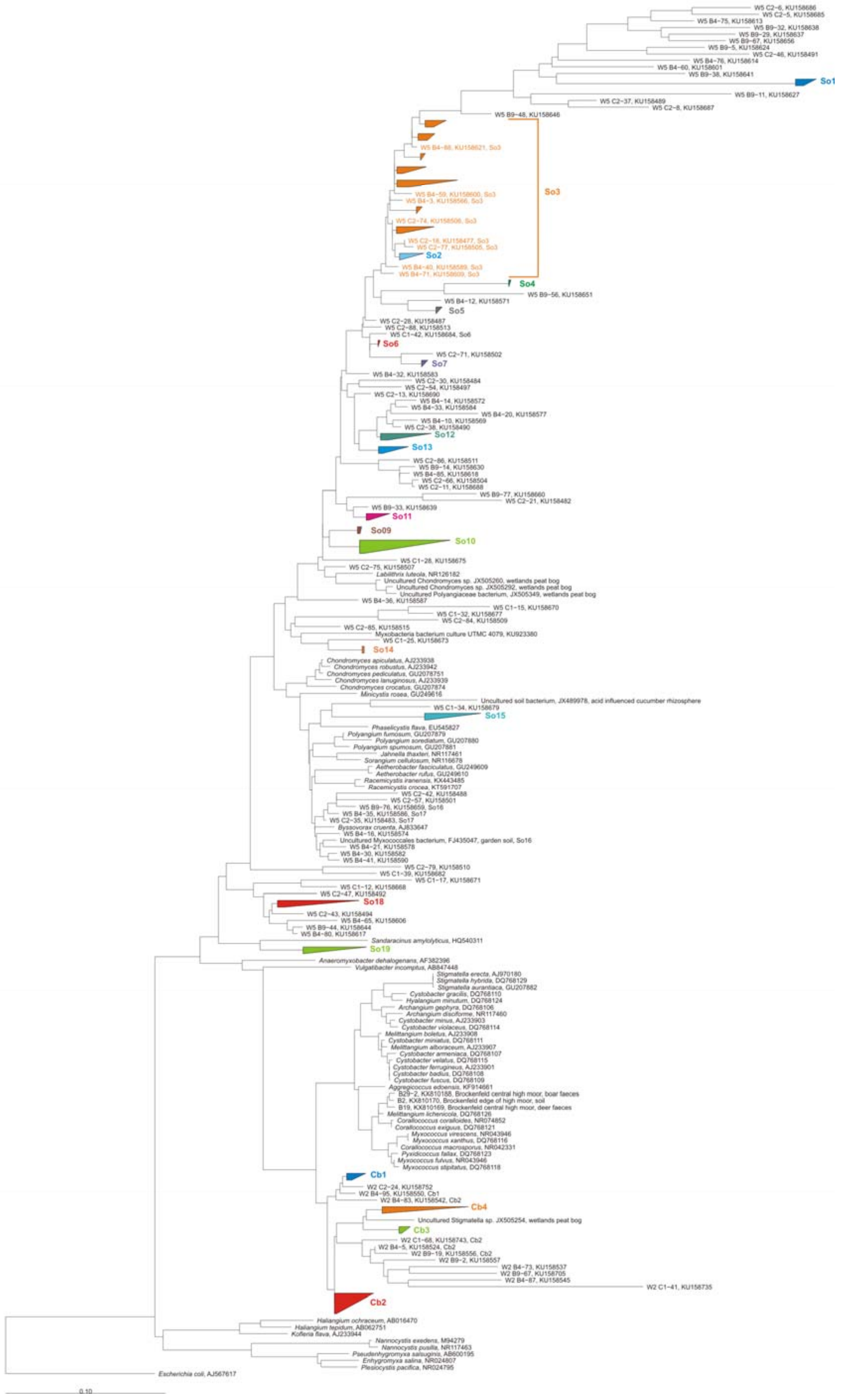
So1
So3
So2
So4
So5
So6
So7
So12
So13
So11
So09
So10
So14
So15
So18
So19
Cb1
Cb4
Cb3
Cb2
0.10

Figure S2. Distance matrix tree based on 16S rRNA-gene sequences of all myxobacterial type strains and clone sequences from the present study, three representatives of cultures from this study (B2, B19, B29-2) as well as sequences from other cultivation-independent studies of acidic soils and one myxobacterial culture (UTMC 4079) from the public database (NCBI). Several OTUs are grouped. W5: forward primer specific for Sorangiineae/Nannocystineae. C1 and C2: sampling site fen Am Sandbeek, soil; B4 and B9: Brockenfeld central high moor, soil. Bar, 0.10 substitutions per nucleotide position.

Isolation of predators with bait bacteria isolated from the moor samples

As an approach to the moor-environment, non-myxobacterial bacteria were also isolated from the samples from which the clone banks have been established and offered as bait. On 1/10 Luria Bertani-agar [per liter: 1.0 g tryptone, 0.5g yeast extract, 1.0 g NaCl; pH 5.5] different dilutions of samples A1, B4, B7, B9, B18, B21, B24, C1 and C2 (in water) were plated and as many morphological different bacteria as possible were isolated. The DNA of the pure cultures was extracted and 16S rRNA gene PCR with primer F27/R1525 was carried out. 16S rRNA genes of bait organisms were partial sequenced with primer F27, F518 and R1100. Consensus sequences were compared with sequences of the NCBI database. Eight different bacteria could be isolated from samples B18, B21, C1, and C2 (Table S2) and were streaked alive cross like on water agar. The moor samples were placed at the end of the cross and incubated at room temperature.

Sample treatment

Water samples were treated in four different ways: From 25 ml centrifuged moor water the pellets were treated in the same way as the solid samples. In addition moor water was diluted 1:1000. A total of 20 µl of this dilution was dropped on water agar/*E. coli* and filter paper (Stan 21). The third approach was plating 100 µl of this dilution on water agar and Stan 21. After drying, E. coli bait and filter pieces were added, respectively. The last approach was filtering 10 ml moor water with a water jet pump through a cellulose filter. The filter was placed on VY/2-agar (per liter: Baker's yeast: 5.00 g, CaCl2 x 2 H2O: 1.36 g, agar (Difco): 20 g, Vitamin B12: 0.50 mg. Sterilized vitamin B12 solution was added after autoclaving separately by filtration).

PCR conditions for amplification of 16S rRNA genes from cultures

A first PCR reaction was started with eubacterial primers F27/R1525 in a volume of 50µl: 25µl JumpStart Taq ReadyMix (Sigma), 0.4 µMol/l of each primer (final concentration), 2µl genomic DNA, 19µl PCR water. PCR amplifications were conducted in a Mastercycler Gradient (Eppendorf) using the following conditions: initial denaturation at 95 °C (5 min); 35 cycles of denaturing at 94 °C (30s); annealing at 52 °C (30s); extension at 72 °C (120s); and a final extension at 72 °C (600s). PCR products were checked on an agarose gel (0.8%), purified using the NucleoSpin® Gel and PCR Clean-up-Kit (Macherey-Nagel) and eluted in 30 µl elution buffer.

Sequence analyses

For a first assignment, 16S rRNA genes of the cultures were sequenced using primer F27 and F518. For six cultivated representatives of the OTUs (C8, B1, C6, C4, B17, C17), full length 16S rRNA genes were sequenced using additionally primer F357 (Muyzer *et al.*, 1993), F945, R1078 and R1525 to assure that both nucleotide directions were covered. The sequences of these six cultures have been deposited at GenBank under accession numbers KP18974-KP18979. The specificity of the two primer combinations (FW2/FW5 and R1525) was checked by PCR using genomic DNA of 21 representatives derived from the phyla Actinobacteria, Cyanobacteria, Firmicutes, α-, β-, and γ-Proteobacteria (Table S1) and additional 22 representatives of the order Myxococcales (Table S2). PCR applicability of extracted DNA from the representatives was initially checked with eubacterial primers F27/R1525 as described for the isolated strains. Afterwards the PCR conditions for the semi-specific myxobacterial primer sets (FW2/FW5 - R1525) were tested. The annealing temperature was optimized using sequential gradient PCR reactions, starting with a temperature range between 50°C and 70°C. The PCR products were checked via agarose gel and the temperature range was narrowed down until an optimal annealing temperature for both primer sets could be defined, such as 61.1°C for combination FW2/R1525 and 65.5°C for FW5/R1525. These conditions were used for the amplification of myxobacterial 16S rRNA genes in the further process.

Data analysis

The 16S rRNA gene sequences acquired via clone bank and from the isolates were checked for quality using the program BioEdit (free available). The sequences from the cultures were assembled into consensus sequences. The single sequences from clones as well as the consensus sequences from cultures were compared with the NCBI database entries. Closely related myxobacterial sequences, representing different species, were imported into the ARB database (version 14.02.2005 database; http://www.arb-home.de) and aligned together with the sequences acquired in this study. A distance matrix tree was constructed with 16S rRNA sequences of 56 myxobacterial type strains using the Neighbour-Joining method (Saitou and Nei, 1987) and Jukes Cantor correction (Jukes and Cantor, 1969). The topology of the phylogenetic tree was built by bootstrap analysis of 1000 operations. As recommended in ARB, no filter was used in bootstrapping. Sequences sharing more than 99% similarity, calculated with the similarity matrix tool in ARB, were grouped into operational taxonomic units (OTUs). For phylogenetic analyses, the 16S rRNA gene of a cultivated representative of each OTU, if there was one, was fully sequenced. Only the type and representative myxobacterial strains as well as clones of uncultured bacteria with highest similarity to the novel isolates were shown in the phylogenetic tree. Table S3 shows the type strains and their corresponding 16S rRNA gene accession numbers used for the construction of the phylogenetic tree. Non Myxococcales-sequences were excluded from our analyses (data not shown). Single sequences or sequences of clones from OTUs which did not include type strain sequences were blasted in GenBank to figure out the next cultivated relative. In all cases, the next cultivated relative belongs to the Myxococcales. So we are sure that all our clone sequences included in this study are of myxobacterial origin.
